# Evaluation of the effects of two doses of alpha glycerylphosphorylcholine on physical and psychomotor performance

**DOI:** 10.1186/s12970-017-0196-5

**Published:** 2017-10-05

**Authors:** Lena Marcus, Jason Soileau, Lawrence W. Judge, David Bellar

**Affiliations:** 1 School of Kinesiology, University of Louisiana at Lafayette, Lafayette, LA 70503 USA; 2 Pennington Biomedical Research Center, Louisiana State University, Baton Rouge, LA 70808 USA; 3 School of Kinesiology, Ball State University, Muncie, IN 47306 USA; 4 School of Kinesiology, University of Louisiana at Lafayette, 225 Cajundome Blvd, Lafayette, LA 70506 USA

**Keywords:** Power, Strength, Reaction time

## Abstract

**Background:**

Recent studies have suggested that alpha glycerylphosphorylcholine (A-GPC) may be an effective ergogenic aid. The present study was designed to assess the efficacy of two doses of A-GPC in comparison to placebo and caffeine for increasing countermovement jump performance, isometric strength, and psychomotor function.

**Methods:**

Forty-eight healthy, college aged males volunteered for the present study and underwent baseline assessment of countermovement jump (CMJ), isometric mid thigh pull (IMTP), upper body isometric strength test (UBIST), and psychomotor vigilance (PVT). Following this assessment participants were randomly assigned to groups consisting of 500 mg A-GPC, 250 mg A-GPC, 200 mg Caffeine or Placebo taken daily. Blood samples were collected 1 h and 2 h post initial dose to quantify serum free choline and thyroid stimulating hormone then subjects returned after 7 days of supplementation to repeat CMJ, IMTP, UBIST and PVT.

**Results:**

No differences were noted between groups for IMTP, UBIST or PVT performance. Serum free choline was found to be elevated in the two A-GPC groups as compared to placebo (132% and 59% respectively). Serum TSH was found to be significantly depressed in the 500 mg A-GPC group compared to other treatments (*p* < 0.04). Group differences were noted for maximum velocity and maximum mechanical power on the CMJ (*p* < 0.05) with the 250 mg A-GPC group demonstrating the greatest improvements in result.

**Conclusions:**

Based upon this evidence, and previous evidence regarding A-GPC, it should be considered as an emerging ergogenic supplement.

## Background

Choline has been the subject of much study in conjunction with human performance [1]. Most of this work has focused on long duration exercise, as choline depletion was thought to play a possible role in fatigue [2]. However, it has been reported that after 4 h of strenuous exercise with either 8.4 g choline citrate (administered prior to and midway through exercise) or placebo there was no effect for either run time to exhaustion or squat tests [3]. Additionally, in another study, no benefit was seen an hour post 2.43 g choline bitartrate administration for exercise to exhaustion at 70 or 150% of VO_2_ max [4]. Based upon the results it seems that if choline could be an effective ergogenic aid for endurance efforts, the exercise would likely need to significantly deplete choline [1]. Another issue that many of these studies have in common is the use of choline salts, which have been demonstrated to be less effective at increasing free choline levels as compared to molecules involved in the choline biosynthetic pathway such a Lecithin (a source of phosphotidylcholine) [5, 6]. This is most likely due to these molecules having a normal function in the synthesis of choline, as opposed to just providing a sources of choline. Another molecule in the choline synthesis pathway is alpha glycerylphosphoryl choline (A-GPC), which similarly was shown to significantly increase plasma free choline levels [7].

Once ingested, A-GPC is converted to phosphorylcholine and can then serve as a source of choline for acetylcholine (ACh) synthesis [8]. A large review of clinical studies on dementia or cerebrovascular disorders suggest favorable results for improved cognitive fucntion with A-GPC compared to control or placebo [9]. ACh is not only a neurotransmitter, but also responsible for the action potential that stimulates a muscle to contract and thus a role for augmenting performance associated with intense muscle contraction both for enhanced power and strength has been suggested for A-GPC [10, 11]. The proposed mechanism would include increased ACh as a result of A-GPC ingestion, leading to a more pronounced signal for contraction that results in increased muscle force production. While this is yet and unproven mechanism two recent studies of nutritional supplements containing 300 mg or 150 mg A-GPC suggested improvements in reaction time and vertical jump power [12, 13]. These studies provided these doses of A-GPC as part of a nutritional supplement to 19 recreational, college aged adults. These individuals then rested for 10 min and were tested on reaction time, power and exhaustive exercise.

The most recent study on A-GPC [11] examined the effects of 600 mg dose on muscle strength. In a group of 13 college aged men, administration of A-GPC resulted in an increase of 98.8 N during an isometric mid thigh pull assessment. The 600 mg dose in this study was similar to that used in previous research that reported positive performance results [10]. One issue with the current research on A-GPC is the variation in doses, with some studies examining 1000 mg [7] and other studies examining A-GPC within a nutritional supplement at a dose of 150 mg [12] and variation in acute vs chronic administration [11–13]. The majority of studies use doses of around 600 mg in an acute fashion, although the only chronic dosing human performance study [11] reported changes in force production. Therefore, it is important to more thoroughly examine the dose – response relationship of A-GPC to determine what is the required dose for improved performance. The purpose of the present investigation was to examine the effects of 2 doses of A-GPC in comparison to a placebo and caffeine for measures of human performance and cognitive function.

## Methods

**Fig. 1:**
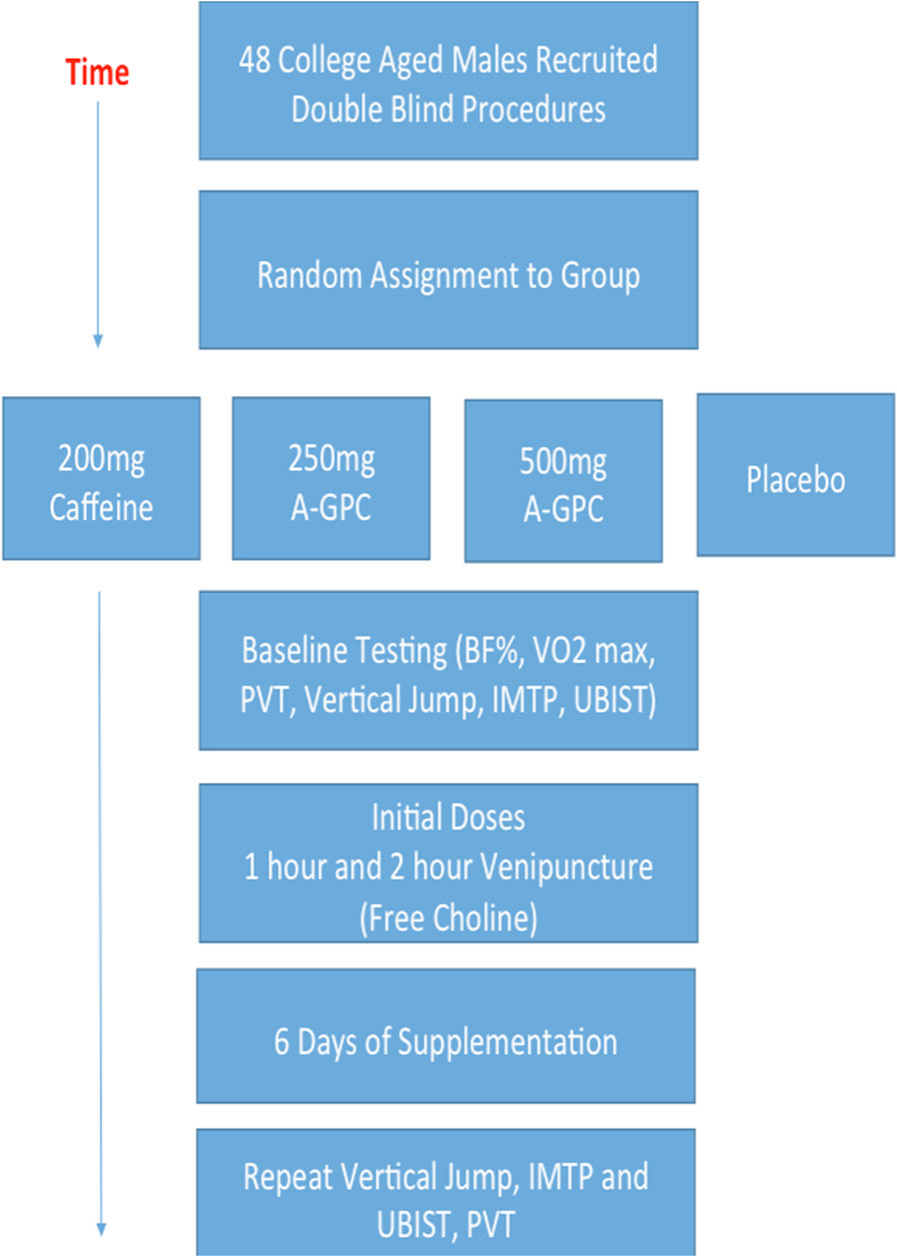
Flowchart of Experimental Procedures

The present study was a double-blind, four arm investigation. The study procedures were reviewed and approved by the Institutional Review Board at the University of Louisiana at Lafayette and all participants gave written informed consent. Four groups of 12 healthy young men volunteered to participate in the study and were randomly assigned to groups (500 mg A-GPC, 250 mg A-GPC, 200 mg Caffeine or placebo). Flowchart of experimental procedures can be seen in Fig. 1.

### Participants

A total of 48 apparently healthy college aged males volunteered to participant in the current investigation (see Table 1). The participants (*n* = 48, height 177.6 ± 7.7 cm, weight 84.2 ± 14.4, BF% 15.3 ± 8.3, VO2 Max 33.8 ± 22.5 ml/kg*min) were recruited from the local University and represent a convenience sample of recreationally trained subjects. The group reported regular engagement in exercise to maintain or enhance fitness. Group means did not differ in major characteristics (*p* > 0.5).

**Table 1:** Participant characteristics

Variable	Group	Mean ± SD	*p* value
Height (cm)	500 mg A-GPC	176.7 ± 8.7	0.64
	250 mg A‐GPC	176.0 ± 6.6	
	200 mg Caffeine	179.7 ± 8.5	
	Placebo	178.9 ± 7.3	
Weight (kg)	500 mg A‐GPC	83.4 ± 14.7	0.64
	250 mg A‐GPC	80.2 ± 8.8	
	200 mg Caffeine	86.0 ± 21.2	
	Placebo	87.3 ± 10.9	
Body Fat%	500 mg A‐GPC	15.3 ± 8.9	0.83
	250 mg A‐GPC	14.7 ± 8.6	
	200 mg Caffeine	13.4 ± 9.1	
	Placebo	16.9 ± 7.5	
VO2 Max (ml/kg*min)	500 mg A‐GPC	33.1 ± 6.3	0.51
	250 mg A‐GPC	34.9 ± 6.2	
	200 mg Caffeine	36.5 ± 5.9	
	Placebo	30.6 ± 4.1	

### Procedures

Participant reported to the lab for the initial visit during the morning hours (0600-0700) after an overnight fast (minimum 8 h), had the experimental procedures explained to them and gave written consent to be part of the study. Afterwards height and weight was measured using a physicians triple beam balance and stadiometer. The participants had an assessment of maximum aerobic capacity via a COSMED CPET system (COSMED, Rome ITL) with integrated electronically braked cycle ergometer and body fat percentage via air displacement plethysmography (Bod Pod Gold Standard System, COSMED Rome, ITL). Following these assessments participants performed 3 baseline trials of a countermovement jump, isometric mid-thigh pull, upper body isometric test and a psychomotor vigilance test. The participants were then assigned to groups and given the first dose of the treatment with 237 ml of cool water (500 mg A-GPC, 250 mg A-GPC, 200 mg caffeine or placebo) and sat quietly in the lab for 120 min post consumption. At 60 min and 120 min post ingestion a blood sample was taken to quantify free choline and thyroid stimulating hormone (TSH). The participants were then given supplements to take for the next 6 days in the morning and were dismissed. On the seventh day the participants returned to the lab fasted having taken their last dose of supplement. The subject then repeated the performance and cognitive measures a second time as outlined above.

### Supplements

The group treatments consisted of two capsules per day for the 7 days of the study. The rational for the number of days of supplementation was based on earlier research on A-GPC [11]. Chemi Nutra (Austin TX) supplied all capsule for the study. The A-GPC capsules (both 500 mg and 250 mg) were supplied with a certificate of analysis from an independent lab, verifying active content through Quantitative nuclear magnetic resonance (NMR). The Caffeine capsule content was verified by an independent lab using high performance liquid chromatography (HPLC). Placebo capsules consisted of microcrystalline cellulose.

### Isometric testing and force plate counter movement jump

The isometric mid-thigh pull test (IMTP) is a well-validated strength measure [14] with reported correlations of 0.5-0.8 with actual competition performance. The test equipment consisted of an AMTI force plate (Advanced Materials Technologies Inc., Watertown MA USA) secured beneath a custom Rouge fitness weightlifting rack (Rogue Fitness Inc., Columbus OH USA). The participants were instructed to stand with the feet shoulder width apart above the force plate. The height of the bar was adjusted so that the participant was in a position where the torso was upright, the knees achieved between 120 and 130 degrees of extension (measured via a goniometer) and the arms were straight while holding the bar. The participants’ hands were secured to the bar via weightlifting hooks to remove the variance associated with grip strength. The participants were told to “drive straight up” and to pull as hard as they could against the bar until the force began to noticeably decline. The peak force was assessed in triplicate at a sampling rate of 2000 Hz using an AMTI Force Plate. The average of the three trials was used for subsequent analysis of variables of interest.

The upper body isometric test (UBIST) used in this study has previously been reported to be both reliable (ICC =0.9) and valid when examined against a 1 RM bench press (*r* = .92) [15]. The participants were positioned on three elevated platforms with the chest directly suspended over a load cell anchored into the concrete floor of the lab (iLoad Pro, Loadstar Sensors, Fremont CA USA). The load cell chosen has a capacity of greater than 5000 Newtons and a listed accuracy of 0.25% for the full scale of measurement. The participants were placed in a push-up style position, with the hands at 150% of biacromial width, and the elbows at 90 degrees of extension (measured via a goniometer). A thick, non-elastic strap was run over one shoulder and under the opposite shoulder and connected with metal rings to a chain that was tethered to the load cell. The participants were positioned so that no slack was apparent in the chain prior to initiation of data capture.

The participants were instructed to keep their backs flat, and push with their hands maximally until told to stop by the researcher. Prior to data capture the load cell was tared to ensure the weight of the load cell and apparatus were accounted for. The researcher started data collection and verbally instructed the participant to “push as hard as possible”. The load cell captured data (150 Hz) until force noticeable declined (drop of 50 N). The average of three trials was used for subsequent analysis of variables of interest. For the countermovement jump (CMJ) the participants were asked to perform three maximum effort jumps off an AMTI Force Plate (Advanced Materials Technologies Inc., Watertown MA USA). For this test the participant placed their hands on their hips, to remove the influence of the upper body. Data from the test was analyzed using a software package specific to CMJ analysis (Flo Inc., Westbrook, ME USA). The average of the three trials was used for subsequent analysis of variables of interest. Data from this study suggested high reliability with ICC values of greater than 0.8.

### Cognitive testing

The Walter Reed palm-held psychomotor vigilance test (PVT) was administered to the participants after resting for at least 10 min in a quiet room. The PVT is a test of simple visual reaction time and was developed at the Walter Reed Army Institute of Research [16] and was used to assess mean reaction time over a 5-min time course. The test used random periods of time in which a target stimulus are displayed on the screen of a Palm handheld device. The program was set to display approximately 100 stimuli in the 300-s (5 min) period at randomly spaced intervals. This program computes a mean reaction time to each stimulus, as well as major and minor lapses in attention. This instrument has been used in large scale studies conducted by the military and is extensively represented as a reliable (test-retest >0.8) cognitive measure in the literature [16].

### Serum free Choline and thyroid stimulating hormone

Blood was collected at 60 and 120 min post initial dose from a vein in the antecubital space into serum separator tubes. The blood was allowed to clot at room temperature and then a clinical centrifuge was used to separate the serum, which was aliquoted and stored at −35 degrees Celsius. At the conclusion of data collection serum aliquots were thawed and free choline levels were determined via a colorimetric assay (BioVision Inc., Milpitas CA USA) read at 450 nm on a microplate reader (ELx 808, Bioteck Winooski VT USA). Similarly, serum TSH was determined by a commercial ELISA kit (Eagle BioScience, Nashua, NH USA).

### Statistical analysis

Differences in baseline performance were assessed via ANOVA. Changes from average baseline performance for IMTP and UBIST were assessed by group via ANOVA. Serum free choline and TSH levels were analyzed via repeated measures ANOVA. Changes in CMJ variable were analyzed via ANCOVA with body mass as a covariate. PVT performance on day seven was examined via ANOVA by group. All statistical analyses were performed using a modern statistical software package JMP 12.0 (SAS Institute Inc. Cary NC USA). Statistical significance was set a priori at *p* < 0.05.

## Results

### Difference in baseline performances

Results of Anova analysis did not reveal any differences in baseline assessments by group for variables associated with isometric measures or counter movement jumps (*p* > 0.41).

### Analysis of isometric measures

IMTP change from pre to post supplementation was examined via ANOVA. A significant treatment effect was revealed via the analysis (F = 2.27, *p* = 0.047). Post-hoc comparison revealed that only the Caffeine treatment was significantly different than the placebo (*p* = 0.036). For the UBIST assessment a treatment effect was not revealed via ANOVA (F = 0.452, *p* = 0.743). See Table 2.

**Table 2:** Changes in isometric measures post-supplementation (M ± SD)

Variable	500 mg A-GPC	250 mg A-GPC	200 mg Caffeine	Placebo
Baseline IMTP Peak Force (N)	3000.01 ± 611.09	2815.49 ± 504.51	3297.14 ± 768.43	3149.82 ± 885.04
Post Supplementation IMTP Peak Force (N)	2927.82 ± 495.66	2972.48 ± 483.67	3515.24 ± 931.61^*^	3024.22 ± 571.99
Baseline UBIST Peak Force (N)	632.21 ± 192.33	632.18 ± 176.29	700.07 ± 221.09	576.66 ± 259.82
Post Supplementation UBIST Peak Force (N)	651.07 ± 189.92	678.52 ± 133.34	715.82 ± 176.78	591.975 ± 268.25

^*^ indicates significantly different than placebo (*p* < 0.05)

### Analysis of countermovement jump measures

For the present investigation average change (pre,post) in mean (trial 1,2,3) maximum force, maximum velocity, maximum impulse, maximum mechanical power and average force were analyzed via ANCOVA (covaried for body mass). No significant differences were revealed for changes in maximum force, average force or impulse. ANCOVA revealed group differences for maximum velocity (F = 0.247, *p* = 0.04) and maximum mechanical power (F = 2.98, *p* = 0.02) during the countermovement jumps. See Table 3.

**Table 3:** Changes in countermovement jump measures post-supplementation (M ± SD)

Variable	500 mg A-GPC	250 mg A-GPC	200 mg Caffeine	Placebo
Maximum Force (N)				
Baseline				
Post Supplmentation				
Change	−69.89 ± 187.35	76.57 ± 195.11	−57.30 ± 306.93	−68.14 ± 195.54
Average Force (N)				
Baseline				
Post Supplmentation				
Change	31.08 ± 61.29	−6.67 ± 34.18	9.24 ± 29.37	19.16 ± 42.59
Maximum Velocity (m/s)				
Baseline				
Post Supplmentation				
Change	−0.12 ± 0.16	0.01 ± 0.13	0.025 ± 0.16	0.00 ± 0.15
Maximum Mechanical Power (Nm/s)				
Baseline				
Post Supplmentation				
Change	−143.83 ± 344.03	151.77 ± 300.54^*^	7.39 ± 309.38	31.29 ± 277.29
Impulse (Ns)				
Baseline				
Post Supplmentation				
Change	69.57 ± 61.29	66.98 ± 10.91	75.48 ± 19.22	73.78 ± 17.36

^*^ indicates significantly different from 500 mg A-GPC *p* < 0.05

### Analysis of psychomotor vigilance testing

ANOVA was used to examine mean reaction time (RT), maximum reaction time (max RT), and minor lapses in attention (>300 msec). The analysis did not reveal any significant differences by group (*p* > 0.5). See Table 4.

**Table 4:** Day 7 Psychomotor vigilance test results (M ± SD)

Variable	500 mg A-GPC	250 mg A-GPC	200 mg Caffeine	Placebo
Mean RT (sec)	0.330 ± 0.059	0.359 ± 0.106	0.315 ± 0.070	0.326 ± 0.070
Max RT (sec)	2.376 ± 3.235	1.351 ± 1.716	2.149 ± 3.914	1.982 ± 3.150
Minor lapes (count)	2.916 ± 1.621	5.000 ± 1.507	2.636 ± 2.292	3.833 ± 5.937

### Serum free Choline and TSH

Analysis of serum free choline via repeated measures ANOVA resulted in a significant difference by treatment (F = 14.98 *p* = 0.001) but not for a treatment by time (1 h, 2 h) interaction (F = 0.196, *p* = 0.928). The caffeine and placebo treatment had the lowest free choline levels respectively, with the 250 mg A-GPC and 500 mg A-GPC demonstrating significantly higher levels (132% and 59% respectively).

Analysis of TSH revealed a significant main effect by group. Post hoc analysis revealed significantly lower TSH levels (500 mg of A-GPC 2.29 ± 0.51μIU/ml, 3.17 ± 1.6 μIU/ml for placebo, 2.97 ± 1.03 μIU/ml for 250 mg of A-GPC and 3.08 ± 0.83 μIU/ml for Caffeine) with the 500 mg A-GPC dose as compared to all other treatment (*p* < 0.04).

## Discussion

The present investigation demonstrated a role for A-GPC in improving power during countermovement jumps and for increasing serum free choline levels. However, these findings require further verification. There have been other studies in the past that have demonstrated increased power [10] and isometric strength [11] with A-GPC supplementation. However, a recent study [17] with a similar design that compared two doses of A-GPC with placebo and caffeine did not demonstrate any significant increases in performance. This study, however, used smaller doses of A-GPC (200 mg, 400 mg) administered acutely 30 min before the performance assessments and additionally did not use forceplate based performance assessments. The data from the present study suggests that 250 mg may represent the minimum dose necessary to see performance changes, however there were great variations in performance amongst the groups. Other studies demonstrating positive results have used doses of 600 mg [10, 11]; therefore this may be the preferred dose at the present with the data available in the literature.

The study also demonstrated that both 250 mg and 500 mg doses of A-GPC were effective at increasing serum free choline, similar to findings reported in an earlier study [7] at a dose of 1000 mg. However, the present study did not demonstrate any changes in psychomotor vigilance performance. Though A-GPC has been shown to improve cognitive function in with persons suffering from vascular dementia [8, 9] these studies have generally used much larger doses of A-GPC (1000-1200 mg daily). Additionally, it is more likely that a study would demonstrate change in cognition with a cognitively compromised population as compared to healthy young adults. Therefore, both the study population and the selected doses may have limited detection of changes in psychomotor vigilance.

However the most promising result form this study may be the depression in circulating TSH levels with 500 mg administration of A-GPC. It is known that increase central dopamine levels reduce TSH levels [18]. A study by Scanlon et al. [18] demonstrated this link through administration of dopamine receptor blockade that lead to significant increases in circulating TSH hormone. Additionally, it has been shown that dopamine infusion inhibits TSH response [19, 20]. In the present study all subjects given the 500 mg dose were found to have TSH levels below 3.0 μIU/ml with the average being 2.29. In a recent publication these subclinical levels were associated with elevated cortisol, a sign of hypothyroidism [21]. This low level of TSH, associated with hypothyroidism, further suggests that the 500 mg dose resulted in a true depression in circulating TSH, possibly caused by increase dopamine. While further exploration of the role of A-GPC in enhancing dopamine is needed to confirm and expand these findings, it suggests a new and exciting area of research. Studies should be conducted in the future to evaluate the potential of A-GPC to enhance dopamine and also cognitive areas such as mood and memory that are effected by dopamine levels.

## Conclusions

Based upon the available evidence from this study, it appears that A-GPC may maintain some ergogenic effects in doses of 250 mg or greater, although lower doses have been found in other studies to offer various degrees of ergogenic effects [10–13]. It can be suggested that athletes and coaches looking to improve performance in events that emphasize velocity and power consider adding A-GPC to their nutritional strategy; however, based upon the total available literature dose of 600 mg or greater are more likely to provide performance results. While more evidence needs to be collected regarding the use of A-GPC, current results are positive particularly in the area of vertical or countermovement jump where a number of studies have been focused. Future research on A-GPC should focus on larger doses for significant performance benefits, while doses lower than this should focus perhaps on other neurological benefits.

## Abbreviations

A-GPC: Alpha glycerylphosphrylcholine
IMTP: Isometric Mid Thigh Pull
TSH: Thyroid Stimulating Hormone
UBIST: Upper Body Isometric Test

## References

[1] Penry JT, Manore MM (2008). Choline: an important micronutrient for maximal endurance-exercise performance?. Int J Sport Nutr Exerc Metab.

[2] Conlay LA, Sabounjian LA, Wurtman RJ (1992). Exercise and neuromodulators: choline and acetylcholine in marathon runners. Int J Sports Med.

[3] Warber JP, Patton JF, Tharion WJ, Zeisel SH, Mello RP, Kemnitz CP, Lieberman HR (2000). The effects of choline supplementation on physical performance. Int J Sport Nutr Exerc Metab.

[4] Spector SA, Jackman MR, Sabounjian LA, Sakkas C, Landers DM, Willis WT (1995). Effect of choline supplementation on fatigue in trained cyclists. Med Sci Sports Exerc.

[5] Wurtman MJ, Hirsch MJ, Growdon JH (1977). Lecithin consumption raises serum free choline levels. Lancet.

[6] Hirsch MJ, Growdon JH, Wurtman RJ (1978). Relations between dietary choline or lecithin intake, serum choline levels and various metabolic indices. Metabolism.

[7] Kawamura T, Okubo T, Sato K, Fujita S, Goto K, Hamaoka T, Iemitsu M (2012). Glycerophosphocholine enhances growth hormone secretion and fat oxidation in young adults. Nutrition.

[8] Parnetti L, Mignini F, Tomassoni D, Traini E, Amenta F (2007). Cholinergic precursors in the treatment of cognitive impairment of vascular origin: Ineffective approaches or need to re-evaluate?. J Neurol Sci.

[9] Parnetti L, Amenta F, Gallai V (2001). Choline alphoscerate in cognitive decline and in acute cerebrovascular disease: an analysis of published clinical data. Mech Ageing Dev.

[10] Zeigenfuss T, Landis J, Hofheins J (2008). Acute supplementation with alpha-glycerylphosphorylcholine augments growth hormone response to, and peak force production during, resistance exercise. J Int Soc Sports Nutr.

[11] Bellar D, LeBlanc NR, Campbell B (2015). The effect of 6 days of alpha glycerylphosphorylcholine on isometric strength. J Int Soc Sports Nutr.

[12] Hoffman JR, Ratamess NA, Gonzalez A, Beller NA, Hoffman MW, Olxon M, Purpura M, Jäger R (2010). The effects of acute and prolonged CRAM supplementation on reaction time and subjective measures of focus and alertness in healthy college students. J Int Soc Sports Nutr.

[13] Shields KA, Silva JE, Rauch JT, Lowery RP, Jäger R, Wilson JM (2014). The effects of a multi-ingredient cognitive formula on alertness, focus, motivation, calmness, and psychomotor performance in comparison to caffeine and a placebo. J Int Soc Sports Nutr.

[14] Beckham G, Mizuguchi S, Carter C, Sato K, Ramsey M, Lamont H, Horsby G, Haff G, Stone M (2010). Relationship of isometric mid-thigh pull variables to weighlifting performance. J Sports Med Phys Fitness.

[15] Bellar D, Marcus L, Judge LW (2015). Validation and reliability of a novel test of upper body isometric strength. J Hum Kinet.

[16] Thorne DR, Johnson DE, Redmond DP, Sing HC, Belenky G, Shapiro JM (2005). The Walter Reed palm-held psychomotor vigilance test. Behav Res Methods.

[17] Parker AG, Byars A, Purpura M, Jäger R (2015). The effects of alpha-glycerylphosphorylcholine, caffeine or placebo on makers of mood, cognitive function, power, speed and agility. J Int Soc Sports Nutr.

[18] Scanlon MF, Weightman DR, Shale DJ, Mora B, Heath M, Snow MH, Lewis M, Hall R (1979). Dopamine is a physiological regulator of thyrotrophin (TSH) secretion. Clin Endocrinol.

[19] Besses GS, Burrow GN, Spaulding SW, Donabendian RK, Pechinski T (1975). Dopamine infusion acutely inhibits the TSH and prolactin response to TRH. J Clin Endo Metab.

[20] Cooper DS, Klibanski A, Ridgway EC (1983). Dopaminergic modulation of TSH and its subunits: in vivo an in vitro studies. Clin Endo.

[21] Walter KN, Corwin EJ, Ulbrecht J, Demers LM, Bennett JM, Whetzel CA, Klein LC (2012). Elevated thyroid stimulating hormone is associated with elevated cortisol in healthy young men and women. Thyroid Res.

